# A Rare Culprit of Spontaneous Abortion, Latent Tuberculosis Complicated by Disseminated Peritoneal TB

**DOI:** 10.1155/2018/2318539

**Published:** 2018-10-28

**Authors:** Christoph Sossou, Chaitanya Pal, Jose R. Bustillo

**Affiliations:** ^1^Resident Physician, Internal Medicine, Newark Beth Medical Center, Department of Medicine, Newark, NJ, USA; ^2^Associate Residency Program Director, Newark Beth Medical Center, Department of Medicine, Newark, NJ, USA

## Abstract

This is a case of a 38-year-old female with latent TB complicated by disseminated peritoneal TB with associated spontaneous abortion, who was initially thought to have an ovarian neoplasm, prompting extensive workup. Laparoscopy with biopsy later confirmed the patient's condition; she was initiated on the appropriate therapy and had a full recovery.

## 1. Case Introduction

Medical awareness of peritoneal tuberculosis (TB) is still lacking, and many women with this disease are initially thought to have ovarian neoplasm [1] and undergo unnecessary extensive workup and, at times, invasive surgical procedures [2]. Gastrointestinal extrapulmonary tuberculosis (TB) is extremely rare [1], and its association with miscarriages has rarely been reported in the literature. This is a case of a 38-year-old Liberian female with untreated latent TB who subsequently developed disseminated peritoneal tuberculosis, complicated by spontaneous abortion.

## 2. Case Description

**Figure 1:**
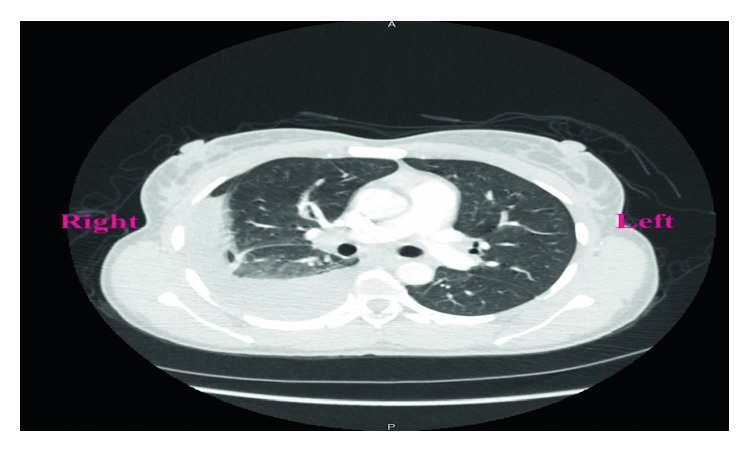
Computed tomography of the chest without contrast. Moderate right pleural effusion with passive atelectasis of the right middle and lower lobes; bilateral hilar lymphadenopathy; perihepatic ascites.

**Figure 2:**
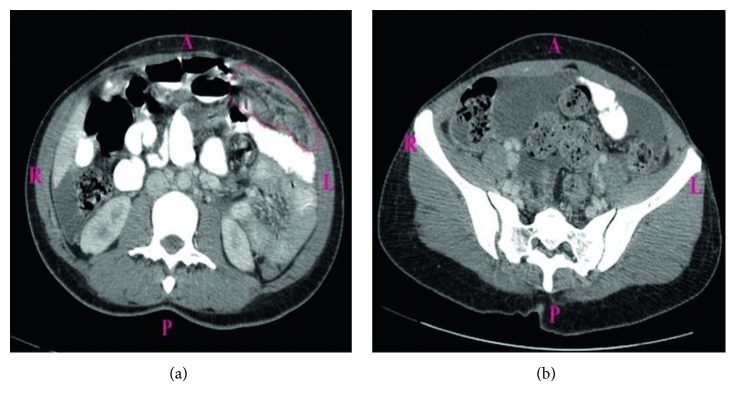
Computed tomography of the abdomen and pelvis with contrast. Diffuse mesenteric enhancement in the left upper quadrant suspicious for carcinomatosis (a) and multiloculated cystic mass adjacent to the right adnexa suspicious for ovarian neoplasm (b).

A 38-year-old Liberian female with a 12-week gestation presented to the emergency department with a 3-week history of low-grade subjective fever, night sweats, unintentional weight loss, gradually worsening abdominal pain, and intermittent spotting. Vital signs were stable on presentation, physical exam noticeable for gravida abdomen, otherwise unremarkable. Laboratory examination revealed beta hCG 118471, which was otherwise unremarkable. Pelvic ultrasound confirmed a 12-week viable intrauterine pregnancy. The patient was admitted to the hospital for close monitoring. Hospital course was complicated by massive pleural effusion, low-grade fever, progressive worsening abdominal pain, and spontaneous abortion. Non-contrast-enhanced computed tomography of the chest (Figure 1) revealed large right-sided pleural effusion, and contrast-enhanced computed tomography of the abdomen and pelvis (Figure 2) revealed bilateral hilar adenopathy, ascites, thickening and enhancement of the peritoneum, and mottled nodular-appearing soft tissue consistent with omental caking suspicious for peritoneal carcinomatosis. She underwent extensive workup including surgical and oncologist consultations for possible exploratory laparotomy and discussion of treatment options for presumed ovarian neoplasm. Blood work revealed elevated carbohydrate antigen (CA) 125 and positive QuantiFERON-TB Gold, but adenosine deaminase, CA 19, alpha-fetoprotein, and inhibin B were within normal limits. Diagnostic laparoscopy with biopsy revealed significant pelvis ascites and diffuse miliary lesions throughout the peritoneum. She underwent dilatation and curettage; histopathologic examination showed chronic granulomatous inflammation with no evidence of neoplasm. Special stains on tissue sections and ascitic fluid stain revealed rare acid-fast bacilli, suggestive of mycobacterial granulomatous peritonitis. Additional questioning indicated a history of positive PPD skin test a year prior without follow-up treatment. The patient was placed on four-drug anti-tuberculous therapy and had a complete recovery.

## 3. Case Discussion

Gastrointestinal extrapulmonary TB is extremely uncommon [2]. Clinical manifestations are nonspecific and protean, but fever, abdominal pain, night sweats, anorexia, weight loss, pleural effusions, and ascites should raise the suspicion of tuberculous peritonitis especially in a patient such as the one in this case report from an endemic area. This is a unique case as it is the first known case in the literature of disseminated peritoneal TB complicated by spontaneous abortion in a patient with history of latent TB. This case raises the vital question of how we should proceed with management of latent TB in women of reproductive age. Our case presents three important points. First, medical awareness of peritoneal TB is lacking [3]. Second, routine diagnostic methods such as direct acid-fast bacilli smear and cultures are of relatively low diagnostic yield in peritoneal TB [3]. Finally, laparoscopy with biopsies is a safe and effective method to diagnose peritoneal tuberculosis [4, 5]. Early diagnosis would aid in avoidance of unnecessary extensive workup and surgery, permitting prompt initiation of appropriate therapy. This would shorten hospital length-of-stay, reduce resource utilization, and increase overall patients' satisfaction.
